# Differential predictors of DSM-5 PTSD and ICD-11 complex PTSD among African American women

**DOI:** 10.1080/20008198.2017.1338914

**Published:** 2017-06-15

**Authors:** Abigail Powers, Negar Fani, Sierra Carter, Dorthie Cross, Marylene Cloitre, Bekh Bradley

**Affiliations:** ^a^ Department of Psychiatry and Behavioral Sciences, Emory University School of Medicine, Atlanta, GAUSA; ^b^ Atlanta VA Medical Center, Atlanta, GAUSA

**Keywords:** Posttraumatic stress disorder, PTSD, complex posttraumatic stress disorder, childhood maltreatment, emotion dysregulation, dissociation

## Abstract

**Background**: Complex posttraumatic stress disorder (CPTSD) is proposed for inclusion in the ICD-11 as a diagnosis distinct from posttraumatic stress disorder (PTSD), reflecting deficits in affective, self-concept, and relational domains. There remains significant controversy over whether CPTSD provides useful diagnostic information beyond PTSD and other comorbid conditions, such as depression or substance use disorders.

**Objective**: The present study examined differences in psychiatric presentation for three groups: traumatized controls, DSM-5 PTSD subjects, and ICD-11 CPTSD subjects.

**Method**: The sample included 190 African American women recruited from an urban public hospital where rates of trauma exposure are high. PTSD was measured using Clinician Administered PTSD Scale for DSM-5 and CPTSD was measured using clinician administered ICD-Trauma Interview. Psychiatric diagnoses and emotion dysregulation were also assessed. In a subset of women (*n* = 60), emotion recognition was measured using the Penn Emotion Recognition Task.

**Results**: There were significant differences across groups on current and lifetime major depression (*p *< .001) and current and lifetime alcohol and substance dependence (*p *< .05), with CPTSD showing the highest rates of comorbidities. CPTSD women also showed significantly higher levels of childhood abuse and lower rates of adult secure attachment. Multivariate analysis of variance showed significantly more severe PTSD and depression symptoms and, as expected, more severe emotion dysregulation and dissociation, compared to DSM-5 PTSD and traumatized control groups. Individuals with CPTSD also had higher levels of emotion recognition to faces on a computer-based behavioural assessment, which may be related to heightened vigilance toward emotional cues from others. CPTSD women had better facial emotion recognition on a computer-based assessment, which may suggest heightened vigilance toward emotional cues.

**Conclusions**: Our results suggest clear, clinically-relevant differences between PTSD and CPTSD, and highlight the need for further research on this topic with other traumatized populations, particularly studies that combine clinical and neurobiological data.

## 1. Introduction

For the upcoming International Classification of Diseases, 11^th^ version (ICD-11), the World Health Organization (WHO) has proposed the inclusion of complex posttraumatic stress disorder (CPTSD) as a new diagnosis that is related to, but separate from, posttraumatic stress disorder (PTSD) (Maercker et al., 2013). A diagnosis similar to CPTSD was first operationalized under the diagnosis of Disorders of Extreme Stress Not Otherwise Specified (DESNOS) for DSM-IV field trials (Roth, Newman, Pelcovitz, van der Kolk, & Mandel, 1997). Although DESNOS was not included in the DSM-IV, the symptom criteria for the proposed ICD-11 CPTSD were selected using data from this the field trial along with data gathered from expert clinicians (Cloitre et al., 2011; van der Kolk, Roth, Pelcovitz, Sunday, & Spinazzola, 2005). The proposed CPTSD disorder requires an ICD-11 PTSD diagnosis but also includes three additional features, including problems in affective, self-concept, and relational domains to meet criteria for the disorder (Cloitre, Garvert, Brewin, Bryant, & Maercker, 2013). In contrast to PTSD symptoms for which negative reactions such as fear and horror are tied directly to trauma-related stimuli, the disturbances in affect, self-concept, and relationships must be shown to be pervasive and occur across a variety of contexts, even in the absence of trauma reminders, and to create significant distress and functional impairment for individuals.

CPTSD is typically (although not exclusively) associated with chronic, repeated traumas, particularly those occurring in early life, such as childhood abuse (Briere & Rickards, 2007; Herman, 1992; Hyland et al., 2017). The concept of CPTSD was first introduced by Judith Herman (Herman, 1992), who described the long-term impact of chronic stress on emotion regulation, self-organization, self-perception, and interpersonal functioning. Relative deficits in these areas of functioning have been identified in research on the impact of childhood maltreatment. Research shows that maltreated children have difficulty with emotional expression, recognition, and reactivity and difficulty in social interactions compared to non-exposed children (Maughan & Cicchetti, 2002; Pollak, Cicchetti, Hornung, & Reed, 2000; Shields & Cicchetti, 2001; Southam-Gerow & Kendall, 2002). These emotional and interpersonal difficulties can persist into adulthood and contribute to many psychological problems (Alink, Cicchetti, Kim, & Rogosh, 2009; Kim & Cicchetti, 2010). This exposure to childhood maltreatment often occurs in combination with insecure attachment to caregivers, reflecting negative internal working models of self and others, which in turn is associated with problems in emotion regulation and interpersonal relationships (Bailey, Moran, & Pederson, 2007; Pearlman & Courtois, 2005). While PTSD (as defined by the ICD-11) remains a core component of CPTSD, the symptoms of CPTSD may be associated with poorer treatment outcomes (Cloitre, Petkova, Su, & Weiss, 2016), and thus identifying the presence of CPTSD may lead to considerations of expanded treatment interventions (Cloitre et al., 2011; Cloitre, Miranda, Stovall-McClough, & Han, 2005).

There remains controversy over the clinical utility of CPTSD as a disorder (Resick et al., 2012; Wolf et al., 2015). Resick et al. (2012) conducted a comprehensive review of research on CPTSD and concluding that data were insufficient to support a distinct diagnostic category from DSM-5 PTSD. One major criticism raised in the review and in the field more broadly is the significant overlap between CPTSD and many other disorders including PTSD, major depressive disorder (MDD), and borderline personality disorder (BPD). It is also possible that CPTSD may be an alternate phenotypic expression of PTSD and not an independent syndrome. Despite these criticisms, however, there is empirical and neurobiological evidence suggesting distinctions between CPTSD and DSM-IV defined PTSD (Lanius, Frewen, Vermetten, & Yehuda, 2010; Lanius et al., 2010; van der Kolk et al., 2005). The diagnostic criteria for PTSD in the DSM-5 (American Psychiatric Association, 2013) has expanded to include four symptom clusters (instead of three). The addition of a negative cognition symptom cluster, an arousal symptom that reflects reckless or self-destructive behaviours, as well as a dissociative subtype were significant changes, and it is possible that this expansion of symptoms may capture some of the same areas of functioning as CPTSD (Friedman, 2013).

To our knowledge, there have not been any empirical studies to date that have examined differences between DSM-5 PTSD and ICD-11 CPTSD. Thus, the goal of the present study was to examine group differences across trauma history and disturbances in attachment, psychiatric disorders, and levels of various psychiatric symptoms among individuals with DSM-5 PTSD and ICD-11 CPTSD in comparison to traumatized control subjects within a sample of African American women with high rates of trauma exposure and psychiatric symptoms. Because women are at significantly higher risk of developing PTSD than men (Breslau, 2001; Olff, Langeland, Draijer, & Gersons, 2007), and urban minority populations are particularly vulnerable to high levels of interpersonal violence and higher rates of PTSD and depression than the general population (Alim et al., 2006; Gillespie et al., 2009), this is an important group with which to study the differences between DSM-5 PTSD and ICD-11 CPTSD. Based on previous research, the psychiatric variables of interest that were tested for differences across groups included childhood abuse exposure, other trauma exposure, adult secure attachment style, diagnostic comorbidities (MDD, alcohol and substance abuse/dependence), and levels of emotion dysregulation and dissociation (which are core components of CPTSD and we expect would be present at high rates in this group of women). Emotion recognition (measured via a computerized task) was also assessed; emotion dysregulation is a core component of CPTSD, and behavioural data capturing emotional awareness of social cues, particularly accurate assessment of emotion in faces, could be informative in further understanding similarities or differences across these groups.

## 2. Method

### 2.1. Procedure

Participants were drawn from an NIMH-funded study of risk factors for the development of PTSD in an urban population. Participants were recruited from waiting rooms in the gynaecology and primary care medical clinics at a publicly funded hospital and the emergency department waiting room of a paediatric, not-for-profit hospital, in Atlanta, Georgia. We did not narrow recruitment to specific selection criteria but approached any individual in the waiting room. To be eligible for participation, subjects had to be 18–65 years old and able to give informed consent. After signing the informed consent approved by the Emory Institutional Review Board, an initial interview was administered by trained research assistants with questionnaires regarding trauma history and psychological variables. More detailed and comprehensive assessments of psychological functioning including PTSD and CPTSD were conducted in a separate associated study (occurring 1–2 weeks after initial assessment); participants were drawn from the pool of participants who completed the initial assessment and were also eligible for other studies being conducted at the lab (see Gillespie et al., 2009, for full details regarding study procedures). Levels of trauma exposure, child abuse severity, PTSD, and depression symptoms were not significantly different between the larger epidemiological sample (*n* = 7636) and the sample for this study (*n* = 190). All participants in the study reported at least one traumatic event that fulfilled Criterion A for a PTSD diagnosis according to the DSM-5 and had completed the comprehensive diagnostic assessment.

Comprehensive diagnostic assessments were conducted in a laboratory setting by trained staff, predoctoral psychology graduate students, and clinical psychology postdoctoral fellows. The training process for the interviewers was rigorous and led by PhD-level psychologists. The process included didactics on relevant DSM-5 disorders, watching numerous videos and in person interviews conducted by PhD-level psychologists (varied by level), and being shadowed for at least two interviews (varied based on individual need). Weekly supervision by PhD-level psychologists was provided during both training and throughout interview administration.

All assessments were conducted in the same order: (1) PTSD, (2) CPTSD, (3) other psychiatric disorders, and (4) self-report measures. Trauma measures and self-reported PTSD and depression symptoms were obtained during the initial assessment.

### 2.2. Participants

The sample consisted of 190 African American women (mean age = 39.39, *SD* = 11.58). The sample was predominately low income, with 63.7% of individuals unemployed and 75.8% coming from households with a monthly income of less than US$2,000. Differences between the groups on demographic variables including age, education level, and household monthly income were assessed using chi-square tests of independence (for categorical or rank order variables) and ANOVA, and no significant differences were found.

### 2.3. Measures

***Clinician-Administered PTSD Scale for DSM-5***(CAPS-5) is an interviewer-administered psychometrically-validated semi-structured diagnostic instrument measuring current DSM-5 PTSD (Weathers et al., 2013). The CAPS-5 was designed to ensure correspondence with DSM-5 and streamline scoring and administration. It measures DSM-5 PTSD symptoms, duration of symptoms, and global impairment and functioning related to symptoms. CAPS-5 yields a continuous measure of the severity of overall PTSD and of the four symptom clusters (re-experiencing, avoidance, negative alterations in cognition/mood, arousal), and presence/absence of PTSD diagnosis and presence/absence of the dissociative subtype. For each diagnostic criterion (20 total), interviewers rate on a scale from 0 (absent) to 4 (extreme/incapacitating) using information on both frequency and intensity of symptoms obtained during the interview. Items with a score of ≥ 2 are counted toward diagnosis. To meet criteria for PTSD based on DSM-5, individuals need to have at least one threshold criterion B symptom (re-experiencing), one threshold criterion C symptom (avoidance), two criterion D symptoms (negative cognitions and mood), and two criterion E symptoms (reactivity and arousal), as well as duration longer than one month and functional impairment present (score ≥ 1). CAPS has been used in both civilian and veteran populations and shown good to excellent reliability and validity across multiple studies (Blake et al., 1995; Bovin et al., 2016; Pupo et al., 2011; Weathers, Keane, & Davidson, 2001). Interrater reliability (IRR) was calculated on a subsample of participants (6.0%, *n* = 12) and showed good IRR for diagnosis of PTSD (*k = *0.83).

***ICD-11 Trauma Interview*** (ICD-TI; Roberts, Cloitre, Bisson, & Brewin, 2013) is an interview-administered diagnostic instrument measuring PTSD and disturbances in self-organization (DSO) which include three symptom domains: affect dysregulation, negative self-concept, and disturbances in relationships (scale: 0–4 based on presence and severity; 0 = absent, 1 = a little bit, 2 = moderately, 3 = very much, 4 = extremely). The self report version of this measure has shown satisfactory reliability (Hyland, Brewin, & Maercker, 2017; Hyland et al., 2017; Karatzias et al., 2016). In order to meet criteria for ICD-11 CPTSD, individuals must meet for ICD-11 PTSD which includes the presence of a threshold rating (≥ 2) on one re-experiencing symptom (i.e. nightmares, flashbacks, or emotional reactivity^1^), one avoidance symptom, and one hyperarousal symptom (i.e. hypervigilance or exaggerated startle response) and meet for each of the three symptom clusters for CPTSD. IRR was calculated on a subsample of participants (6.0%, *n* = 12) and showed excellent IRR for diagnosis of CPTSD (*k = *1.00). Recent research provides empirical support for distinct PTSD and CPTSD profiles in the self report version of this measure (Karatzias et al., 2017; Murphy, Elklit, Dokkedahl, & Shevlin, 2016; Perkonigg et al., 2016).

***Childhood Trauma Questionnaire*** (CTQ; Bernstein et al., 2003) is a 25-item, reliable and valid self-report instrument assessing abuse and neglect in childhood with specific subscales for sexual, physical, and emotional abuse. Based on established scores by Bernstein and Fink (Bernstein & Fink, 1998), a categorical variable was created to account for presence/absence of exposure to moderate-to-severe emotional (score ≥ 13), physical (score ≥ 10), and sexual (score ≥ 8) abuse in childhood (0 = none/mild abuse; 1 = moderate/severe abuse scores for ≥ one type of abuse), and continuous severity scores for overall exposure to abuse.

***Traumatic Events Inventory*** (TEI) is a 14-item screening instrument for lifetime history of traumatic events, assessing type and frequency of trauma(s) experienced. Consistent with prior research (Gillespie et al., 2009; Schwartz, Bradley, Sexton, Sherry, & Ressler, 2005), total level of trauma exposure was measured by a sum score reflecting the number of trauma types (e.g. serious accident or injury, sexual assault) to which a participant had been exposed in their lifetime (excluding child abuse).

***Adult Attachment Prototype Questionnaire***(AAPQ). Based on data gathered during clinical interviews, participants were rated by research interviewers using the AAPQ (Westen, Nakash, Thomas, & Bradley, 2006) for degree of match to descriptions of secure, anxious, avoidant, and disorganized/unresolved adult attachment style prototypes through a 5-point Likert rating. Prior research found predicted relationships between this instrument and adaptive functioning, psychiatric symptoms, and developmental history variables supporting its validity. Both prior research using the AAPQ (Westen et al., 2006) and recent data from our research group indicate strong interrater reliability for this measure (intraclass *r* ^2^= 0.76). Presence/absence of secure attachment was primary interest for present study (*n* = 78, 44.1%).^2^

***MINI International Neuropsychiatric Interview***(MINI; Lecrubier et al., 1997; Sheehan et al., 1997) is a well-validated structured interview developed to assess psychiatric disorders according to the DSM-IV-TR diagnostic criteria. This measure was used to assess current and lifetime presence of MDD and current and lifetime presence of alcohol and substance abuse/dependence.

***Modified Posttraumatic Stress Disorder Symptom Scale***(MPSS; Coffey, Dansky, Falsetti, Saladin, & Brady, 1998) is an 18-item self-report measure assessing DSM-IV-TR PTSD symptoms. This measure has shown good reliability and validity (Coffey et al., 1998). Internal consistency in this sample was high (α = 0.92).

***Beck Depression Inventory-II*** (BDI-II; Beck, Steer, & Brown, 1996) is a widely used, 21-item self-report measure of depressive symptoms. Multiple studies have shown good reliability and validity for the BDI-II (Beck et al., 1996). In the present study, internal consistency of the BDI scale was high (α = 0.93).

***Difficulties in Emotion Regulation Scale*** (DERS) is a 36-item psychometrically-validated (Gratz & Roemer, 2004) self-report measure of emotion regulation difficulties. It includes six subscales measuring different aspects of emotion regulation: (1) non-acceptance of emotions, (2) difficulty engaging in goal-directed behaviour in the presence of negative emotions, (3) difficulty controlling impulses in the presence of negative emotions, (4) lack of awareness of emotions, (5) limited use of effective emotion regulation strategies, and (6) lack of understanding of emotions. Internal consistency of the DERS total scale was high (α = 0.82).

***Multiscale Dissociation Inventory*** (MDI) is a 30-item self-report measure of general dissociative symptomatology experienced during the previous month (Brière, 2002). It measures six different types of dissociative response, including disengagement, depersonalization, derealization, emotional constriction, memory disturbance, and identity dissociation. The MDI has shown good psychometric properties in both normative and validation samples (Brière, 2002; Briere, Weathers, & Runtz, 2005). Internal consistency of the MDI scale was high (α = 0.91).

***Penn Emotion Recognition Task*** (ER40; Gur et al., 2002; Kohler, Turner, Gur, & Gur, 2004; Kohler et al., 2004) is a computerized measure of emotion recognition that was run as part of a larger neuropsychiatric battery on a subset of participants. Participants view a series of 40 faces and are asked to determine which emotion the face is showing for each trial (anger, fear, happy, sad, or neutral). Faces were balanced for equality and intensity of emotion, age, gender, and ethnicity (Kohler et al., 2004).^3^ This measure has shown good reliability and validity and has been normed in both healthy and psychiatric adult samples (Gur et al., 2001, 2010; Irani et al., 2012). Number of correct responses was used as the variable of interest for the present study, including overall correct response (mean = 32.15, *SD* = 3.97, range = 18–37).

### 2.4. Data analysis

The overall analytic approach was to examine differential rates of trauma exposure, adult attachment style, psychiatric disorders, and levels of various psychiatric symptoms by group (i.e. traumatized control, DSM-5 PTSD, and ICD-11 CPTSD). Individuals included in the DSM-5 PTSD group met criteria for DSM-5 PTSD, but not ICD-11 CPTSD.^4^ Individuals included in the ICD-11 CPTSD group met criteria for both ICD-11 PTSD and ICD-11 CPTSD. Although this was not a requirement, within this sample all individuals included in the CPTSD group also met for DSM-5 PTSD despite differences in diagnostic criteria for ICD-11 PTSD and DSM-5 PTSD. See Table 1 for descriptive details of trauma variables, psychiatric diagnoses, and symptoms in the overall sample. Differences across groups in rates of psychiatric disorders and presence of adult secure attachment were assessed using chi-square tests of independence. A multivariate analysis of variance (MANOVA) was conducted to examine differences across continuous variables of interest by group. Since ER40 task data were available for only a subset of participants (*N* = 60),^5^ a separate ANOVA was conducted to assess mean differences in emotion recognition accuracy across the three groups. All analyses were conducted with SPSS 23.0 software package. There was no missing data for continuous variables of interest; all measures were completed by all participants with the exception of adult attachment style (*n* = 177) and the emotional face recognition task (*n* = 60).

**Table 1.** Descriptive details of trauma variables, psychiatric diagnoses, and symptoms in overall sample.

Psychiatric diagnoses and symptoms	*N*	%
Moderate-to-severe exposure to abuse	86	45.0
DSM-5 PTSD	72	38.1
ICD-11 CPTSD^a^	33	17.4
	**Mean (*SD*)**	**Range**
Child abuse severity (CTQ)	43.04 (19.56)	25–108
Lifetime trauma load (TEI; excluding child abuse)	4.37 (2.74)	0–12
PTSD symptom severity (mPSS)	15.44 (12.39)	0–47
Depression symptom severity (BDI)	16.14 (12.04)	0–65
Total emotion dysregulation symptoms (DERS)	70.63 (23.10)	36–141
Non-acceptance	10.92 (5.09)	
Difficulty with goal-directed behaviour	11.37 (5.04)	
Difficulty controlling impulses	11.15 (5.24)	
Lack of emotional awareness	12.90 (4.94)	
Difficulty with emotion regulation strategies	14.66 (5.96)	
Lack of emotional clarity	9.63 (4.00)	
Total dissociative symptoms (MDI)	48.58 (18.30)	30–110
Disengagement	10.28 (4.47)	
Depersonalization	6.63 (2.86)	
Derealization	8.17 (3.68)	
Emotional constriction	9.09 (4.39)	
Memory disturbance	8.14 (3.46)	
Identity dissociation	6.27 (2.97)	

CTQ = childhood trauma questionnaire; TEI = traumatic events inventory; mPSS = modified PTSD symptom scale; BDI = Beck Depression Inventory; DERS = difficulties in emotion regulation scale; MDI = multiscale dissociative index; ^a^all individuals that met criteria for CPTSD also met criteria for DSM-5 PTSD.

## 3. Results

Prevalence of psychiatric disorders was compared across groups. Table 2 shows all significant differences across groups by psychiatric diagnoses. Across all psychiatric diagnoses assessed, the CPTSD group showed the highest diagnosis rates, both currently and over the lifetime. Additionally, 36.4% (*n* = 12) of individuals in the CPTSD group also met criteria for the dissociative subtype based on DSM-5 criteria, while only 5.4% (*n* = 2) of individuals in the DSM-5 PTSD only group met criteria for the dissociative subtype.

**Table 2.** Psychiatric descriptive details compared across groups.

	Traumatized control	DSM-5 PTSD	ICD-11 CPTSD	
	*n = *118	*n = *39	*n = *33	
	*N* (%)	*N* (%)	*N* (%)	Overall *p*-value
Current major depression	12 (10.16)	11 (28.2)^a^	22 (66.67)^bc^	*p *< .001***
Lifetime major depression	47 (39.83)	29 (74.36)^a^	28 (84.85)^b^	*p *< .001***
Current alcohol abuse or dependence	6 (5.01)	5 (12.82)	6 (18.18)^b^	.04*
Lifetime alcohol abuse or dependence	23 (19.49)	12 (30.77)	16 (48.48)^b^	.002**
Current substance abuse or dependence	7 (5.93)	2 (5.13)	9 (27.27)^bc^	< .001***
Lifetime substance abuse or dependence	26 (22.03)	18 (46.15)^a^	18 (54.55)^b^	< .001***

Differences in rates of psychiatric disorders between individuals in the three groups were assessed using chi-square tests of independence. Significant differences are depicted with asterisks: *p < .05, **p < .01, ***p < .001. For differences across groups: ^a^denotes significant difference (*p* < .05) between traumatized control and PTSD only groups; ^b^denotes significant difference between traumatized control and CPTSD groups; ^c^denotes significant difference between PTSD and CPTSD groups.

**Figure 1.**
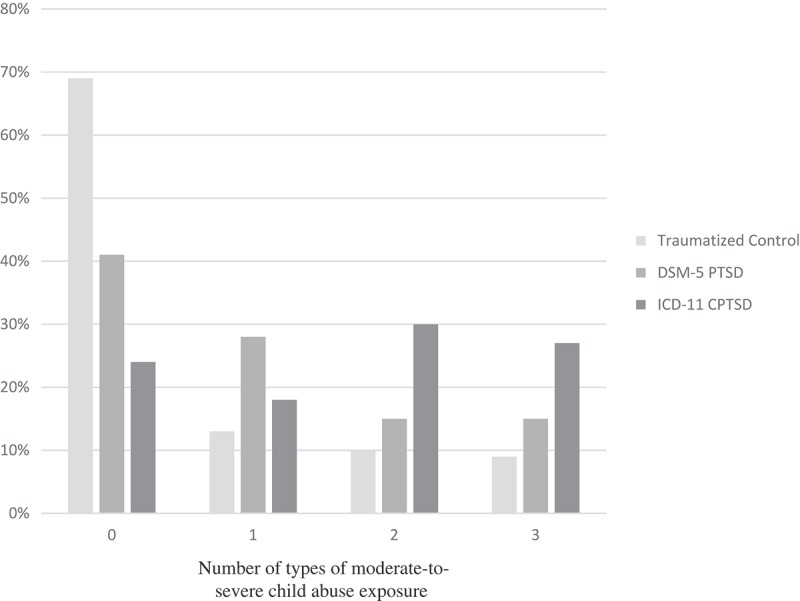
Percentage of number of types of exposure to moderate-to-severe childhood emotional, physical, and/or sexual abuse by group type.

The multivariate ANOVA examining psychiatric variables by group was significant (Wilks’ Lambda *F* = 5.09, *p* < .001, partial η^2^ = 0.36), and all variables of interest were significantly different across groups (see Table 3). Posthoc LSD analyses yielded significantly higher scores for the CPTSD in almost all areas compared with the traumatized control and DSM-5 PTSD groups. Regarding trauma exposure, the ICD-11 CPTSD group was significantly higher in level of child abuse compared with traumatized controls and DSM-5 PTSD groups (*p* < .01; see Figure 1). As shown in Figure 1, the ICD-11 CPTSD group were more likely to report the experience of multiple types of moderate-to-severe abuse than the DSM-5 PTSD or control groups. However, DSM-5 PTSD and ICD-11 CPTSD groups were not significantly different in overall trauma load (excluding child abuse). Individuals with CPTSD also showed significantly lower rates of adult secure attachment than both PTSD and traumatized control groups (CPTSD: 9.10%, DSM-5 PTSD: 33.33%, traumatized controls: 52.54%, *p* < .05). Additionally, the ICD-11 CPTSD group had significantly higher levels of all dimensions of emotion dysregulation and dissociation, with the exception of DERS emotional awareness. A separate MANCOVA was also run to evaluate whether these effects remained when MDD diagnosis was included as a covariate in analyses; the overall model remained significant independent of current MDD (Wilks’ Lambda *F* = 3.17, *p* < .001, partial η^2^ = 0.27). There were no significant differences in outcomes, with the exception of DERS emotional awareness no longer being significantly different across groups.

**Table 3.** Multivariate analysis of variance predicting psychiatric symptoms and outcomes by group (traumatized control, DSM-5 PTSD, and ICD-11 CPTSD).

	Traumatized control	DSM-5 PTSD	ICD-11 CPTSD		
	*n = *118	*n = *39	*n = *33		
	Mean (*SD*)	Mean (*SD*)	Mean (*SD*)	*F*	*Partial Eta Squared*
Total child abuse severity (CTQ)	38.11 (17.41)	46.86 (20.00)^a^	56.12 (19.84)^bc^	13.42***	0.13
Number of abuse types experienced (CTQ)	0. 58 (0.98)	1.05 (1.10)^a^	1.61 (1.14)^bc^	13.41***	0.13
Overall trauma load (excluding child abuse, TEI)	3.60 (2.52)	5.35 (2.97)^a^	5.97 2.19)^b^	14.55***	0.14
Current depressive symptoms (BDI)	11.65 (10.17)	18.52 (8.57)^a^	29.35 (11.38)^bc^	41.07***	0.31
Current PTSD symptoms (mPSS)	9.63 (8.88)	20.97 (10.12)^a^	29.67 (11.24)^bc^	64.56***	0.41
Emotion dysregulation symptoms (DERS)	62.31 (19.63)	76.96 (21.35)^a^	92.89 (19.65)^bc^	32.63***	0.26
Non-acceptance of emotion (DERS)	9.52 (4.10)	12.10 (4.93)^a^	14.52 (6.38) ^bc^	15.92***	0.15
Difficulty with goal-directed behaviour (DERS)	9.63 (4.36)	12.51 (4.29)^a^	16.27 (5.16)^bc^	31.27***	0.25
Difficulty managing impulses (DERS)	9.63 (4.18)	12.51 (4.29)^a^	15.33 (5.83)^bc^	19.55***	0.17
Lack of awareness of emotions (DERS)	12.25 (4.49)	13.46 (4.98)	14.53 (6.02)^b^	3.12*	0.03
Difficulty with regulation strategies (DERS)	12.65 (4.89)	16.41 (5.92)^a^	19.80 (5.93)^bc^	26.07***	0.22
Lack of clarity of emotions (DERS)	8.63 (3.58)	10.26 (4.00)^a^	12.45 (4.02)^bc^	14.16***	0.13
Dissociation symptoms (MDI)	41.41 (12.52)	50.90 (14.74)^a^	71.50 (20.25)^bc^	55.64***	0.37
Disengagement (MDI)	8.75 (3.53)	10.62 (4.06)^a^	15.36 (4.21)^bc^	39.96***	0.30
Depersonalization (MDI)	5.70 (1.53)	6.59 (2.67)^a^	10.00 (4.05)^bc^	41.61***	0.31
Derealization (MDI)	6.72 (2.44)	9.13 (3.30)^a^	12.21 (4. 44)^bc^	44.23***	0.32
Emotional constriction (MDI)	7.58 (3.47)	9.85 (3.67)^a^	13.58 (4.884)^bc^	33.31***	0.26
Memory disturbance (MDI)	7.10 (2.87)	8.59 (2.92)^a^	11.31 (4.00)^bc^	24.23***	0.21
Identity dissociation (MDI)	5.55 (1.95)	6.13 (1.82)	9.03 (4.95)^bc^	21.66***	0.19

*p < .05, ***p < .001.

Posthoc analyses were computed using LSD: ^a^denotes significant difference (*p* < .05) between traumatized and PTSD groups; ^b^denotes significant difference between traumatized control and CPTSD groups; ^c^denotes significant difference between PTSD and CPTSD groups.

A separate ANOVA was conducted to examine mean differences in correct responses on an emotion recognition task (*F*_60_ = 3.36, *p* < .05, partial η^2^ = 0.11). On this task, the CPTSD group showed a significantly higher number of correct responses in comparison to both the traumatized control and DSM-5 PTSD groups (*p *< .05; traumatized control: mean = 31.54, *SD* = 4.47; PTSD: 31.69, *SD* = 2.81; CPTSD: mean = 35.00, *SD* = 1.33).

## 4. Discussion

This study provides a novel examination of differences in the psychiatric presentation of individuals with DSM-5 PTSD and ICD-11 CPTSD in comparison to those without PTSD within a sample of traumatized African American women. CPTSD is a proposed diagnosis for the ICD-11, and there remains a great deal to understand about its value in providing unique and useful information above and beyond a DSM-5 PTSD diagnosis alone. Our results suggest that there are clinically relevant differences between DSM-5 PTSD and CPTSD that can inform treatment. In particular and in support of previous research (Briere & Rickards, 2007; Herman, 1992; Roth et al., 1997), individuals exposed to multiple types of child abuse were at increased risk for the development of CPTSD. CPTSD was also associated with less likelihood of having secure attachment, and more comorbidity with other psychiatric conditions. It is possible that some of the psychiatric symptoms that were also higher in the CPTSD group, such as emotion dysregulation and dissociation, may act as risk factors for multiple comorbid psychiatric conditions or create more difficulty with day-to-day functioning in the presence of these conditions. Targeting these broader symptoms in the context of treatment may thus aid in promoting greater treatment success. It is important to note that the differences found between groups was present independent of MDD diagnosis and these differences reflect symptoms not captured by PTSD and MDD alone, suggesting the potential utility of CPTSD as an independent construct. Given the cross-sectional nature of our study, it is impossible to determine the causal pathways between symptoms and disorders. Additional longitudinal research will be critical to examine what mechanisms may be contributing to risk for psychiatric conditions in traumatized women.

When specifically examining the dimensions of emotion dysregulation, there were important similarities and differences between the PTSD and CPTSD group. The CPTSD group had higher overall emotion dysregulation scores, as well as higher scores on all but one dimension of emotion dysregulation (non-acceptance of emotions). This finding suggests that teaching acceptance or tolerance of negative emotions could be important in treating all individuals with PTSD symptoms, regardless of CPTSD symptoms. Importantly, all other areas of emotion dysregulation were higher in the CPTSD group compared to DSM-5 PTSD and traumatized controls, further highlighting that emotion dysregulation is a strong and central component of CPTSD. The DSM-5 PTSD group did show significantly higher rates of emotion dysregulation compared to traumatized controls as well, supporting the evidence that emotion dysregulation is a key element of PTSD. However, the significantly higher levels in the CPTSD group demonstrate that emotion dysregulation is an area of deficit for these women that far exceeds what is found in DSM-5 PTSD alone. Indeed, one of the symptoms of CPTSD is affect dysregulation, either via hyperactivation or deactivation, and our findings support a more generalized deficit in emotion dysregulation, rather than one that is particular to only one or two dimensions. These differences highlight ways within which individuals with CPTSD may struggle to understand their emotional reactions and implement strategies to combat strong emotions, making it difficult to manage day-to-day tasks and follow through with treatment.

Individuals with CPTSD also showed higher general dissociation (across all six dimensions of dissociation measured), as well as higher incidence of the symptoms required for a diagnosis of the DSM-5 dissociative subtype for PTSD. Previous research from our laboratory with the same population of women suggests that dissociation may be a maladaptive form for emotion regulation (Powers, Cross, Fani, & Bradley, 2015). Thus, these symptoms of dissociation may also benefit from treatment promoting adaptive emotion regulation abilities. There is growing evidence to suggest that emotion-focused treatment can benefit individuals with PTSD (Cloitre, 2009; Cloitre, Koenen, Cohen, & Han, 2002) and, although both PTSD and CPTSD groups may benefit from focused treatment to improve emotion regulation skills, this focus, based on the findings of the current study, may be even more relevant for women with CPTSD. It is important to note that not all women with CPTSD also met for DSM-5 dissociative subtype, and therefore the clinically relevant differences between the CPTSD women and the women with DSM-5 PTSD would otherwise be missed using the current diagnostic approach in DSM-5.

Interestingly, a relative advantage for facial emotion recognition was observed in a behavioural task for individuals with CPTSD. Compared to traumatized controls and those with PTSD, the CPTSD group had better performance on a task of facial emotion recognition, suggesting that these women may more accurately interpret social emotional cues even though they report poor clarity and understanding of their own emotions. Given that the traumatized controls’ performance was similar to the PTSD only group, these findings may indicate that these superior emotion recognition abilities are unique to CPTSD. Some research on maltreated children has shown that, compared to non-maltreated children, those exposed to childhood abuse are more sensitive to, or more vigilant for, social emotional cues, particularly those signifying threat (e.g. fearful face) (Masten et al., 2008). This may be an adaptive cognitive processing style in the presence of ongoing threat; rapid and accurate assessment of social threat cues may help the maltreated child avoid further harm. However, the persistence of a heightened vigilance or sensitivity toward social emotional cues may lead to increased rumination on these cues. In patients with CPTSD, perceptions of emotion in others may occupy an inordinate amount of cognitive resources in those with CPTSD, which is likely to disrupt many facets of functioning.

It is important to note that we did not see an overall difference in trauma exposure, outside of childhood abuse, among our PTSD and CPTSD groups, further supporting past research showing the particular effect of childhood trauma on the development of CPTSD (Briere & Rickards, 2007; Cloitre et al., 2009; Roth et al., 1997). Herman (1992) and others have proposed that CPTSD can develop from chronic, interpersonal trauma even in the absence of exposure to childhood abuse (e.g. domestic violence). Although it is beyond the scope of the present study, more specific examination of the impact of type of adult traumatic experiences, duration of trauma, and age of exposure on the development of CPTSD would be beneficial as researchers continue to move forward in understanding the aetiology of this disorder.

It is also certainly possible that insecure attachment, even in the absence of significant child abuse exposure, may contribute to risk for CPTSD. We found that the CPTSD group was significantly less likely to show secure attachment in adulthood than DSM-5 PTSD or traumatized control groups. This finding is notable considering the research showing the negative impact insecure attachment can have on functioning and treatment outcomes (Pearlman & Courtois, 2005; Stalker, Gebotys, & Harper, 2005) and may be important to keep in mind when considering potential differential treatment implications for individuals with CPTSD. It should be noted that rating of adult attachment state can be indicative of the type of attachment style developed during childhood, but specific childhood attachment variables were not assessed in the present study.

Several study limitations are worth noting. First, given the cross-sectional nature of this study and the use of retrospective reports, we cannot make assertions about causality or time of onset for PTSD, CPTSD, other psychiatric symptoms, emotion dysregulation, or dissociative symptoms. Additionally, our use of a sample of low socioeconomic traumatized African American women may limit generalizability of these findings to other populations. However, this potential limitation is counterbalanced by the public health importance of studying these variables in an often under-researched and under-served population characterized by lower economic resources, repeated traumatic exposure, and high incidence of psychopathology, often undiagnosed (Gillespie et al., 2009; Schwartz et al., 2005). This is a useful population in which to study the long-term effects of child abuse as there remains limited data on the long-term impact of trauma among these women and such research could provide critical information that might improve treatments specifically for such women.

Overall, these findings suggest that the proposed ICD-11 CPTSD diagnosis does reflect clinically significant differences from DSM-5 PTSD diagnosis, and is related to higher levels of child abuse exposure and comorbid adult psychiatric disorders. The relatively high levels of emotion dysregulation and general dissociative symptoms may increase these individuals’ vulnerability to the onset of other psychiatric conditions, and could be a particular target for treatment in those with CPTSD. The differences in trauma presentation, attachment, emotion dysregulation, and dissociation are present independent of MDD, supporting the argument that CPTSD may be a distinct construct and not solely a representation of more severe PTSD and comorbid MDD. Because the presence of symptom profiles reflecting variants of complex PTSD may negatively impact treatment success (Cloitre et al., 2016) and benefit from additional treatment approaches (Cloitre et al., 2011), this remains a very important area of study as the field moves forward in trying to improve the success of trauma-related treatments.

## Notes

1. Emotional reactivity was only counted as a re-experiencing symptom if the trauma queried occurred in childhood and included childhood physical or sexual abuse or witnessing violence within the home.

2. Only 177 of the 190 participants had completed AAPQ data; participants were not significantly different from those that did not complete task on demographic or trauma exposure variables.

3. Only 60 of the 190 participants completed the neuropsychological task; participants were not significantly different from those that did not complete task on demographic or trauma exposure variables.

4. Eighty-four percent of individuals in the DSM-5 PTSD group also met criteria for ICD-11 PTSD (*n* = 64).

5. For this subgroup, the breakdown across groups was as follows: traumatized control, *n* = 37; PTSD only, *n* = 13, PTSD + CPTSD, *n* = 10.

## Highlights

- This is the first study to examine differences between DSM-5 PTSD and ICD-11 complex PTSD among traumatized civilian African American women
- Looking across traumatized control, DSM-5 PTSD, and ICD-11 complex PTSD groups, significant differences in trauma exposure, attachment style, and psychiatric symptomology were found between DSM-5 PTSD and ICD-11 complex PTSD
- Complex PTSD may be important as a distinct diagnosis from PTSD and valuable for informing treatment and improving intervention success in highly traumatized populations
